# Quality of life and its related factors in women with substance use disorders referring to substance abuse treatment centers

**DOI:** 10.1186/s12905-020-01155-7

**Published:** 2021-01-06

**Authors:** Majid Barati, Khadijeh Bandehelahi, Tahereh Nopasandasil, Hanieh Jormand, Amir Keshavarzi

**Affiliations:** 1 Social Determinants of Health Research Center, Hamadan University of Medical Sciences, Hamadan, Islamic Republic of Iran; 2 Asadabad School of Medical Sciences, Asadabad, Islamic Republic of Iran; 3 Department of Public Health, School of Health, Hamadan University of Medical Sciences, Hamadan, Islamic Republic of Iran; 4 Students Research Committee, Hamadan University of Medical Sciences, Hamadan, Islamic Republic of Iran; 5 Department of Psychiatry, Research Center for Behavioral Disorders and Substances Abuse, School of Medicine, Hamadan University of Medical Sciences, Hamadan, Islamic Republic of Iran

**Keywords:** Substance abuse, Women, Quality of life

## Abstract

**Background:**

Substance-Related Disorders are among the most common social problems caused by using legal and illegal substances. Therefore, this study aimed at determining the quality of life (QoL) and its related factors among women with substance use disorders referring to substance abuse treatment centers in Hamadan, west of Iran.

**Methods:**

This cross-sectional study was carried out on 120 Iranian female substance users recruited through the census sampling method in 2018. Data collection tools consisted of demographic characteristics and QoL assessment (SF-36). Data were analyzed using SPSS-16 via one-way analysis of variance (ANOVA) and chi-square tests.

**Results:**

The mean age of the participants was 33.2 ± 12.1 years and the mean score of their total QoL was 35.35 ± 13.5. The results of multiple linear regression analysis indicated that using methamphetamine (β =  − 6.62) was the predictor of QoL in women. Moreover, there was a significant association between QoL and age (*p* < 0.001), educational level (*p* = 0.011), and age at first use (*p* < 0.001).

**Conclusion:**

According to the results, the participants’ QoL was found to be at an unsatisfactory level. So, it is essential to implement educational help-seeking behavior for treatment and effectiveness educational, as well as holding mental health intervention, school-based substance abuse prevention, and harm reduction programs of substance use. This is especially important in adolescents, young, low-educated, early drug use, and methamphetamine user women, as it may increase the QoL

## Background

Substance abuse is one of the most common social issues and problems [1, 2]. According to the definition proposed by the World Health Organization (WHO), addictive substances include every substance that the complications resulting from its consumption can affect the physical and psychological health of the individual and family, as well as the economic, social, political, and cultural systems of the community [3, 4].

According to the World Drug Report of 2019, the number of Drug Trafficking has increased [2]. Perhaps, drug use had previously been a problem exclusively observed in males; however, due to the departure from the traditional lifestyle, the growth of urbanization, and females’ social movements, women are also similarly subjected to social phenomena such as substance abuse [5]. Based on the statistical data on population published by the United Nations (2017), about 271 million people aged 15–64 years old form about 5% of the world’s total population practice substance abuse. Globally, in 2019, about 35 million people are estimated to suffer from drug use disorders and who require treatment services, according to the latest World Drug Report (UNODC) [2]. Although addiction seems to be a male issue in Iran, women as half of the population are directly and indirectly affected by drug use disorders [6]. In general, statistical data indicate that women account for 9% of all drug-dependent women, quadrupled over the past decade [7]. Various studies have investigated the etiology of addiction and drug-dependence among women. Based on the results of such studies, several factors including the availability of substance, lack of awareness, pressure from spouses or friends, the need for a detachment from reality, poverty, domestic violence, sexual abuse, psychological anxiety, the presence of drug-dependent persons in the family, and divorce are the reasons for drug use in females [8, 9].

The use of narcotic substances by females has negative consequences and outcomes such as abandonment, the formation of an addicted generation, violence, self-mutilation, tattooing as risk factors for transfusion-transmitted diseases [10], shared injection, unprotected sexual behavior, and reduced communication with ordinary individuals [11]. According to studies conducted in Iran, of all women with a drug dependence problem, 5–17% have a history of unprotected sexual relationships. Moreover, hepatitis C is observed in 1.9–100%, tattooing in 35.7%, the use of shared syringes for injection in 45%, sexually transmitted infections such as syphilis in 1–6%, chlamydia in 1–5%, herpes in 38–61%, and HPV in 42% of female drug users [12]. Since the most prevalent HCV transmission mode is injecting drugs with unclean needles or syringes, intravenous drug users are the most crucial group who should be considered [13]. However, drug-dependent women share needles and syringes with partners who have high-risk sexual behaviors [12]. According to a study, 78.1% of women with substance use disorder problems report repeated sexual abuse and 82.1% reported repeat physical abuse [14]. However, most women with drug dependence and drug use disorders conceal their problems as they fear stigma and discrimination [11, 15]. Nowadays, improving the quality of life (QoL) is one of the most important goals of treatment intervention programs. In general, QoL is defined as an individual’s perception of his/her health status and the degree of satisfaction with that condition. The WHO defines the QoL as a person’s perception of his/her status in life associated with goals, expectations, values, and individual concerns. Existing evidence suggests an undesirable QoL among Iranian drug-dependent women [16]. Also, it is generally confirmed that an Iranian female population is a high-risk group that did not satisfy their QoL [17]. It has been evidenced that self-assessed addiction to crack is strongly and negatively associated with all SF-36 subscales [18].

Since women are responsible for motherhood and the next generation’s upbringing, the presence of a drug-dependent mother in the family can cause serious harm to the spouse and children and, consequently, to the community [19]. Therefore, it is essential to design and implement comprehensive interventions to prevent substance use in women. Furthermore, to devise effective educational programs and interventions to improve the QoL of drug-dependent women, it is essential to obtain information about the status of using drugs and the related changes in the QoL. Hence, the present study aimed at determining the QoL and its related factors among drug-dependent women covered by substance abuse treatment centers in Hamadan.

## Method

The present study was a cross-sectional study conducted on women referring to Hamadan’s substance abuse treatment centers in 2018. The present study was carried out on 120 Iranian female substance users recruited through census sampling methods. After identifying and listing substance abuse treatment centers in Hamadan that exclusively provided females’ services, the samples were selected from all women referring to the centers using the census methods.

After coordinating with the authorities and obtaining their approval, the researcher visited the substance users and invited them to participate in the study.

Data gathering in the present study was performed in a way that to achieve diversification by distributing questionnaires at various days and times in the multi-centric, high traffic substance abuse treatment centers to complete questionnaires. Moreover, all willing women could fill out questionnaires and participate in the study inclusion–exclusion criteria.

The researcher ensured the volunteers about the confidentiality of the research and collected data on QoL. The inclusion criteria of the study were recruiting only drug-dependent people who were living in the city or suburbs of Hamadan, had a history of substance use in the past or present, were willing to take part in an interview or complete a questionnaire, and had a history of referring to public substance addiction treatment centers exclusively designed for women. Based on the exclusion criteria, the researchers excluded individuals visiting private centers and were unwilling to cooperate with the research team. In this study, data collection tools consisted of one standard questionnaire and one checklist that collected data on the status of substances and quality of life. After obtaining informed consent, the questionnaires were completed using interviews and self-reports by the participants.

Next, demographic data, including age, life status, education level, and job status, were obtained from the participants. Also, the status of substance use checklist was used to collect data on the type of substance, including cannabis, opium, heroin, cocaine, ecstasy, and new industrial substances used by the subjects within the past one month, six months, and one year. Each item was investigated using a separate question, answered with Yes or No [20].

Questionnaire on the QoL with 36 questions (SF-36): this questionnaire has 36 items categorized into eight subscales. These subscales are physical functioning (10 questions), role impairment due to physical health/role physical (4 items), role impairment due to emotional health/role emotional (3 items), energy and fatigue/vitality (4 questions), mental health (5 items), social functioning (2 questions), body pain (2 items), and general health (5 questions). Two other general subscales are achieved by integrating the subscales known as Physical Component Summary (PCS) and Mental Component Summary (MCS). In this questionnaire, low scores represent a lower QoL, and vice versa [21]. In this regard, a previous study indicated that the SF-36 tool produced reliable data on the health status of people with substance use disorder [18].

The collected data were analyzed by SPSS 23 using descriptive statistics (mean, standard deviation, etc.) and linear regression tests to determine the predictors of QoL. Statistical analysis was performed at a significance level of 0.01.

## Results

In this study, 120 drug-dependent women participated, among those referring to treatment centers during data gathering. Twenty participants were excluded from the study due to incomplete filling of the questionnaire.

The age range of the participants was between 15 and 73 years, with a mean age of 33.2 ± 12.1 years. Of these participants, 33.3% were in the age group of 26–35 years and 30.8% were in the age group of 15–25 years. Other demographic data are presented in Table 1.

**Table 1:** Characteristics of the studied participants (n = 120)

Variables	Category	N	Percentage (%)
Age	15–25	37	30.8
	26–35	40	33.3
	36–45	25	20.9
	46–55	10	8.3
	> 55	8	
Level of education	Illiterate	6	5
	Elementary education	29	24.2
	Secondary education	62	51.7
	Diploma	19	15.8
	University education	4	3.3
Living condition	With parent	7	5.8
	With father	3	2.5
	With mother	6	5
	Lonely	47	39.2
	With family	57	47.5
Job status	Jobless	73	60.8
	College student	4	3.3
	Student	6	5
	Worker	22	18.3
	Farmer	1	0.8
	Employee	3	2.5
	Free job	8	6.7
	Other	3	2.5

**Fig. 1:**
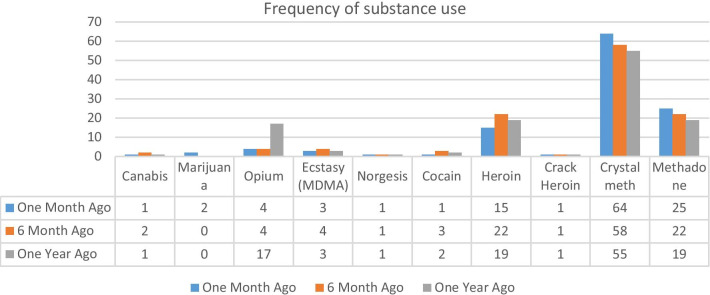
Frequency of substance use within the past one and six months and one year among the studied participant

The most used drugs by the study participants, which were used in the past one month, were crystal, methadone, and heroin, with a prevalence of 53.3%, 20.8%, and 12.5%, respectively. Moreover, the most frequently used drugs by the drug-dependent women, which were used in the past six months, were crystal, methadone, and heroin, with a prevalence of 48.3%, 18.3%, and 18.3%, respectively. The most frequently used drug in the past year was crystal, with a prevalence of 45.8%. The most common drug used by the studied participants over their life span was opium and its derivatives (burnt, sap, etc.) such that 64.2% of them have used it. Crystal, methadone, and heroin use were reported by 53.3%, 49.2%, and 40% of the participants, respectively (Fig. 1).

Based on the obtained results, the participants achieved only 35.3% of the maximum achievable score of the total QoL, indicating the low QoL among the drug-dependent women in Hamadan. Moreover, considering the pain and discomfort score results, the participants obtained 38.3% of the maximum achievable scores, which indicates an unfavorable status. The participants obtained a score of 8.75 in the subscale of physical function (role physical), which was the minimum score among all the subscales and indicated the most favorable condition. Table 2 presents the scores of all subscales of the QoL among the studied people.

**Table 2:** Mean, SD, and range of scores and percentage of mean from maximum obtainable score for dimensions of life quality

QOL dimensions	Mean	SD	Range	Percentage (%)
General health	34.79	17.2	0–100	34.7
Physical functioning	36.07	25.2	0–100	36.1
Role physical	8.75	25.4	0–100	8.75
Bodily pain	38.27	21.6	0–100	38.3
Social functioning	44.06	21.1	0–100	44
Mental health	10.56	26.2	0–100	10.5
Vitality	35.79	19.2	0–100	35.8
Role emotional	41.17	14.5	0–100	41.1
Total score of QOL	35.35	13.5	0–100	35.3

Besides, there was a statistically significant relationship between the women’s QoL with age (*p* < 0.001), educational level (*p* = 0.011), and age at first use (*p* < 0.001). Besides, there was a statistically significant difference based on the post hoc analyses with Tukey tests in 15–25, 46–55, and > 55 years women with Elementary and diploma level of Educations and 10–15, 16–20, and 26–30 years women at First Use. The results showed that QOL scores were lower in adolescent and young, low educated, and early drug use women (Table 3).

**Table 3:** Association between QOL with demographic variables in study participants

Variables	QOL		The significance level
	Mean (SD)	N	
*Age*			
**15–25**	**26.88 (9.96)**	37	F = 4.85 P < 0.001^**^
26–35	28.78 (8.94)	40	
36–45	34.87 (13.51)	25	
**46–55**	40.51 (21.87)	10	
** > 55**	42.46 (21.5)	8	
*Level of education*			
Illiterate	24.57 (3.71)	6	F = 3.46 P = 0.011^*^
Elementary education	**25.61 (6.23)**	29	
Secondary education	32.36 (15.80)	62	
Diploma	36.58 (9.72)	19	
University education	42.75 (21.04)	4	
*Age at first use*			
**10–15**	**25.66 (7.74)**	70	F = 13.57 P < 0.001^**^
**16–20**	38.77 (16.31)	23	
21–25	34.75 (6.79)	6	
**26–30**	41.22 (17.18)	21	
*Living condition*			
With parent	35.02 (19.35)	7	F = 0.78 P = 0.54
With father	38.25 (29.88)	3	
With mother	36.98 (8.06)	6	
Lonely	29.60 (11.58)	47	
With family	31.388 (13.80)	57	
*Job*			
Jobless	32.11 (14.9)	73	F = 1.72 P = 0.11
College student	26.96 (5.73)	4	
Student	27.61 (4.95)	6	
Worker	26.25 (7.27)	22	
Farmer	32.26 (0)	1	
Employee	24.4 (6.36)	3	
Free job	41.1 (17.91)	8	
Other	40.82 (1.39)	3	

**The level of significance was set at P < .01, *The level of significance was set at P < .05

The National Drug Strategy framework suggested several drug types that cause the most harm. This classification includes alcohol, tobacco, cannabis, methamphetamines (e.g., MDMA), and other stimulants such as cocaine, new psychoactive substances (e.g. synthetic drugs, opioids, including heroin, the non-medical use of prescription drugs) [22]. Based on this framework, we classified substances into five categories of “cannabis category included marijuana, cannabis; opium category included opium, heroin, methadone, tramadol and, crack; the non-medical use of prescription drugs such as Norjizak; methamphetamine category included ecstasy (MDMA) and crystal; and cocaine. The present analysis of crack’s chemical combination showed that this substance in Iran is a heroin-based narcotic that is different from the cocaine-based crack used in Western countries [23].

Based on the simple linear regression analysis results, the methamphetamine category (B =  − 6.62) was identified as the predictor of the QoL of the women who participated in the study (Table 4).

**Table 4:** Linear regression analysis to predict the QOL base on substance abuse

Independent variables	B	SE	β	95% CI		*P* value
				Lower	Upper	
Cannabis category	− 5.50	2.89	− 0.17	− 11.21	0.22	0.059
Opium category	7.21	4.66	0.14	− 2.01	16.44	0.12
Methamphetamine category	− 6.62	2.44	− 0.24	− 11.45	− 1.79	0.008^**^
Cocaine category	− 9.82	13.61	− 0.06	− 36.48	17.41	0.49

**The level of significance was set at P < .01

β, standardized regression coefficient; SE, standard error; CI, confidence interval

## Discussion

This study aimed to determine the QoL and its related factors among drug-dependent women covered by substance abuse treatment centers in Hamadan. According to the study results, the participants’ age at first abuse of different materials ranged between 16 and 25 years, with a mean age of 23.57 ± 1.54. These findings indicate that the vulnerability to substance use is higher among people at a young age [24]. Therefore, it can be concluded that the probability of drug use increases in this age range. Undoubtedly, women in the mentioned age range experience severe stress and distress and may consider drug use to reduce stress. As observed in the results, the most frequently used drugs by the participants in their lifetime were opium and its derivatives, crystal, methadone, and heroin. This finding is in line with the results reported by Rahimi-Movaghar et al. [7].

The findings of this study indicate an undesirable QoL among the drug-dependent women in Hamadan. Consistent with our research, Muller et al. reported the poor status of life quality among drug-dependent women [25]. In another study by Tracy et al. in the United States, low QoL was reported among drug-dependent women [26].

The development of tolerance and physiological and psychological dependence on addictive substances can lead to irritability, aggression, and other psychological symptoms. In general, this process reduces physical functioning, undermines psychosocial capabilities, and decreases individuals’ QoL. Generally, QoL is a function of several factors such as the mental health dimension. In the current study, this dimension was lower compared to the other dimensions. However, mental state disorders usually encompass those psychiatric disorders, such as mood, anxiety, and substance use disorders [27]. Thus, personality disorder commonly co-occurs with mental state disorders, causing enormous consequences of substance use disorders. Several studies have shown that mental state disorders are associated with an increased risk of low QoL in physical, psychological, and social domains [28]. Therefore, mental state disorders have been associated with significant impairment in QoL [27, 29]. Accordingly, it is necessary to conduct an analytical study including a case group (women with substance use disorder) and a control group (healthy women). It is also necessary to design and implement educational interventions to improve the QoL of these women.

In the present study, age, education, and age at first drug abuse were associated with women’s QoL. As observed, the QoL was lower among drug-dependent women who were younger, had lower education levels, and started drug abuse at younger ages. The results of this study are consistent with those of other studies. For example, Marini et al. reported a relationship between the QoL of people with a history of substance use and their level of education [30]. In another study, Muller et al. showed that the QoL in illiterate women and those with a low education level was unfavorable [25]. Sadeghi et al. showed that implementing therapeutic interventions in young women and those who started taking drugs at an early age helped to improve different dimensions of QoL one, four, and eight months after the treatment [31]. Therefore, it is necessary to design mental health intervention, school-based prevention, and harm reduction programs of substance use interventions for drug-dependent women who are younger, have a low level of education, and started taking drugs at an early age.

In the present study, the use category of methamphetamine was identified as the predictor of drug-dependent women’s QoL. Findings of a study by Mihan et al. showed negative impacts on their global functioning and QoL among methamphetamine users. In this regard, the evidence suggests a high prevalence of attention-deficit hyperactivity disorder (ADHD) in adults who take methamphetamine. Also, QoL scores were significantly lower in those with ADHD and duration of methamphetamine use [32]. Hence, it is vital to design interventions for drug-dependent women who are methamphetamine users.

This study had some limitations, as it was conducted only on women with a history of drug use referring to substance abuse treatment centers in Hamadan. Thus, it is recommended to conduct similar studies on other drug-dependent women who do not refer to substance abuse treatment centers. Self-reporting and the use of a cross-sectional design were among the other limitations of the study. Due to this study design, causality could not be demonstrated. So, it is suggested to conduct qualitative studies to explain the reasons for women’s addiction and explain the low QoL in women who use substances. Examination variance in the severity of harm with Substance Consumption level and purity of substance investigations in participation is another limitation of our study. Thus, it is suggested to perform preclinical toxicology and observational assessment to understand the damages related to the QoL. A multi-centric data gathering method was used in this study. As indicated, all women present in treatment centers during data gathering were recruited in the study. As a result, the sample size is small and it does not represent all the women with substance use disorder population for generalizability. As the strength of this study, age, education, early drug abuse, use of methamphetamine category, and cocaine category are essential factors that were used in identifying QOL in women with substance use disorder population. Also, applying the SF-36 questionnaire for evaluating QOL in drug-dependent women may confirm the reliability of using this questionnaire even in such a sample.

## Conclusion

According to the results, the participants’ QoL was found to be an unsatisfactory level. So, implementing educational help-seeking behavior for treatment and effective educational, mental health intervention, school-based substance abuse prevention, and harm reduction programs of substance use is essential. This is especially important in adolescents and young, low-educated, and early drug use, and methamphetamine user women, as it may increase their QoL.

## Data Availability

All supporting data are available through the corresponding author.

## Abbreviations

PCS: Physical Component Summary
MCS: Mental Component Summary
QoL: Quality of life
